# Evolution of parasite transmission dispersion

**DOI:** 10.1098/rsos.240629

**Published:** 2025-01-22

**Authors:** Hannelore MacDonald, Sebastian Bonhoeffer, Roland Regoes

**Affiliations:** ^1^ Institute for Integrative Biology, ETH Zürich, 8005 Zürich, Switzerland

**Keywords:** superspreading, parasite, evolution, epidemiology

## Abstract

An open question in epidemiology is why transmission is often overdispersed, meaning that most new infections are driven by few infected individuals. For example, around 10% of COVID-19 cases cause 80% of new COVID-19 cases. This overdispersion in parasite transmission is likely driven by intrinsic heterogeneity among hosts, i.e. variable SARS-CoV-2 viral loads. However, host heterogeneity could also indirectly increase transmission dispersion by driving parasite adaptation. Specifically, transmission variation among hosts could drive parasite specialization to highly infectious hosts. Adaptation to rare, highly infectious hosts could amplify transmission dispersion by simultaneously decreasing transmission from common, less infectious hosts. This study considers whether increased transmission dispersion can be, in part, an emergent property of parasite adaptation to heterogeneous host populations. We develop a mathematical model using a Price equation framework to address this question that follows the epidemiological and evolutionary dynamics of a general host–parasite system. The results predict that parasite adaptation to heterogeneous host populations drives high transmission dispersion early in epidemics. Furthermore, parasite adaptation can maintain increased transmission dispersion at endemic equilibria if virulence differs between hosts in a heterogeneous population. More broadly, this study provides a framework for predicting how parasite adaptation determines transmission dispersion for emerging and re-emerging infectious diseases.

## 1. Introduction

Transmission events are often overdispersed during epidemics, meaning that the majority of infections are transmitted from a minority of infected individuals [1]. For example, less than 20% of cases cause 80% of new infections in typical outbreaks of measles and COVID-19 [2,3]. This increased dispersion in parasite transmission is likely driven to a considerable extent by intrinsic biological heterogeneity among hosts [4,5]. Consequently, the majority of studies on transmission dispersion focus on the direct impact of host heterogeneity [3,5,6]. However, host heterogeneity could also indirectly enhance transmission dispersion by generating selection pressure for parasites that are more transmissible on some hosts than others.

Parasite adaptation to heterogeneous host populations could lead to increased transmission dispersion. In natural populations, differences in symptomatic responses to large within-host pathogen densities can make some hosts more infectious than others [5,7,8], e.g. asymptomatic children have significantly higher SARS-CoV2 viral loads than hospitalized adults [9]. Previous work has shown that distinct host types select for pathogens with different virulence levels (pathogen-induced mortality), which decreases the transmission of evolved pathogens infecting novel host types [10,11]. Heterogeneous host populations composed of individuals whose infectiousness and morbidity vary following infection could drive increased transmission dispersion if pathogens evolve to specialize on hosts that drive more onward transmission. For example, pathogen adaptation could reduce the proportion of infections responsible for most new cases if pathogens evolve high within-host growth rates to exploit hosts supporting high pathogen densities despite low mortality, which simultaneously decreases transmission in other host types by driving high mortality following infection.

This article begins by outlining a simple epidemiological model to demonstrate how host heterogeneity alone contributes to transmission dispersion. We then show how parasite adaptation could create a situation where transmission is increasingly dominated by a few, highly efficient host–parasite interactions using the Price equation framework developed in [12]. The advantage of this approach is that the epidemiological and evolutionary dynamics occur on the same time scale (in contrast to other approaches such as adaptive dynamics) [13]. The framework thus predicts how parasites adapt throughout epidemics rather than only providing predictions at epidemic equilibria.

The framework used here predicts that host heterogeneity can increase transmission dispersion both directly and indirectly through its impact on parasite adaptation. More specifically, parasite adaptation to heterogeneous host populations can drive high transmission dispersion early in epidemics and can maintain increased transmission dispersion at endemic equilibria. In addition, large differences in host quality are predicted to select for parasites that drive high transmission dispersion. This article not only highlights the complex interplay between host heterogeneity and parasite evolution but also provides a framework for predicting how parasite adaptation determines transmission dispersion for emerging and re-emerging infectious diseases.

## 2. Methods

### 2.1. How host heterogeneity impacts transmission dispersion

We first introduce a basic epidemiological model with two distinct host types to demonstrate how host heterogeneity impacts parasite transmission dispersion in the absence of parasite evolution. The heterogeneous host population is composed of two host types with distinct transmission and virulence functions, both of which depend on the within-host growth rate of the parasite (ϵ). Thus, parasites with identical within-host growth rates transmit and increase host mortality at different rates in the two host types. This is modelled by assuming that susceptible and infectious hosts that are ‘high yield’ from the perspective of the parasite ($s_{H},i_{H}$) have high transmission and/or low virulence while infected, such that parasite reproductive fitness from these hosts is high. Conversely, hosts that are ‘low yield’ from the perspective of the parasite ($s_{L},i_{L}$) have low transmission and/or high virulence following infection, such that parasite reproductive fitness from these hosts is low. The within-host growth rate of the parasite is constant in this first model, but will be the trait under selection in the model that includes parasite adaptation. The epidemiological dynamics are given by


(2.1*a*) $$\frac{ds_{H}}{dt}=\lambda (1-p)-({\bar{\beta }}_{H}i_{H}(t)+{\bar{\beta }}_{L}i_{L}(t))s_{H}(t)-\delta s_{H}(t),$$

(2.1*b*) $$\frac{ds_{L}}{dt}=\lambda p-({\bar{\beta }}_{H}i_{H}(t)+{\bar{\beta }}_{L}i_{L}(t))s_{L}(t)-\delta s_{L}(t),$$

(2.1*c*) $$\frac{di_{H}}{dt}=({\bar{\beta }}_{H}i_{H}(t)+{\bar{\beta }}_{L}i_{L}(t))s_{H}(t)-(\delta +\gamma +{\bar{\alpha }}_{H})i_{H}(t),$$

(2.1*d*) $$\frac{di_{L}}{dt}=({\bar{\beta }}_{H}i_{H}(t)+{\bar{\beta }}_{L}i_{L}(t))s_{L}(t)-(\delta +\gamma +{\bar{\alpha }}_{L})i_{L}(t),$$

where λ is the rate that new susceptible hosts enter the system—a proportion p of which are low yield and a proportion (1−p) of which are high yield—δis the natural host mortality rate and γ is the rate that hosts recover from infection. ${\bar{\alpha }}_{j}$ is the additional mortality suffered by infected hosts (virulence) and ${\bar{\beta }}_{j}$ is the average transmission rate in each host type (j=H,L). Both ${\bar{\alpha }}_{j}$ and ${\bar{\beta }}_{j}$ are functions of the within-host growth rate of the parasite (ϵ).

The transmission and virulence functions differ between high- and low-yield hosts such that parasites with the same within-host growth rate trait value will transmit and increase host mortality at different rates in the two host types. In line with previous theory on the evolution of parasite virulence [14,15], the model assumes a trade-off between transmission and virulence such that transmission (${\bar{\beta }}_{j}$) and virulence (${\bar{\alpha }}_{j}$) functions increase as ϵ increases. The function for the transmission rate is


(2.2) $${\bar{\beta }}_{j}(\epsilon ,c_{j},x,\rho )=\rho (c_{j}+\epsilon^{x}),$$

where x controls the concavity of the transmission function as the within-host growth rate increases, ρ is a scaling parameter and $c_{j}$ (j=H,L) is the transmission set point which can vary between high and low-yield hosts. We assume that to study how concave trade-offs between transmission and within-host parasite growth rates impact selection. $c_{j}\geq 0$ and is higher in high-yield hosts when transmission varies between the two host types. Note that transmission would occur even when $c_{j}>0$ and ϵ=0 (no parasite); however, this is not a practical concern in this study as evolutionary analyses always assume positive starting values of ϵ and selection never drives ϵ to 0 (§3).

The function for virulence is


(2.3) $${\bar{\alpha }}_{j}(\epsilon ,y_{j})=y_{j}\epsilon ,$$

where $y_{j}$ is the rate that virulence increases as the within-host growth rate increases. $y_{j}$ is higher in low-yield hosts when virulence varies between the two host types.

The expected number of new infections produced by an infected host is defined following [16] and [17] to study how host heterogeneity impacts parasite fitness and transmission dispersion over time:


(2.4) $$R_{e}(t)=\frac{{\bar{\beta }}_{H}s_{H}(t)}{\delta +\gamma +{\bar{\alpha }}_{H}}+\frac{{\bar{\beta }}_{L}s_{L}(t)}{\delta +\gamma +{\bar{\alpha }}_{L}}.$$

Transmission dispersion can then be defined as the variance-to-mean ratio (following Lloyd-Smith *et al.* [6]) in $R_{e}$ from each host type


(2.5) $$vmr(R_{e}(t))=var(R_{e}(t))/R_{e}(t),$$

where


(2.6) $$\begin{array}{rl} var(R_{e}(t))= & \sum_{j}i_{j}(t)/(i_{H}(t)+i_{L}(t))(R_{e}(t)-R_{ej}(t){)}^{2}\\ = & i_{H}(t)/(i_{H}(t)+i_{L}(t))(R_{e}(t)-\frac{{\bar{\beta }}_{H}(s_{H}(t)+s_{L}(t))}{\delta +\gamma +{\bar{\alpha }}_{H}}{)}^{2}\\ & +i_{L}(t)/(i_{H}(t)+i_{L}(t))(R_{e}(t)-\frac{{\bar{\beta }}_{L}(s_{H}(t)+s_{L}(t))}{\delta +\gamma +{\bar{\alpha }}_{L}}{)}^{2},\\ & R_{ej}(t)=\frac{{\bar{\beta }}_{j}s_{j}(t)}{\delta +\gamma +{\bar{\alpha }}_{j}}\\ & j=H,L. \end{array}$$

**Figure 1.**
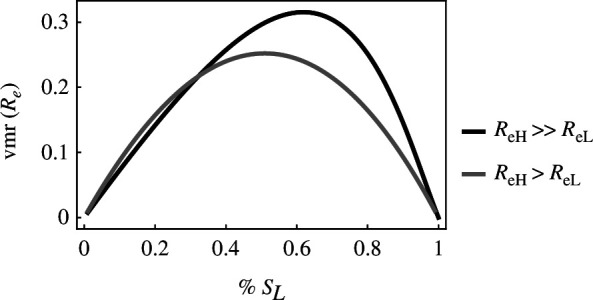
Large differences in host quality drive increased transmission dispersion ($vmr(R_{e})$). Transmission dispersion is high when the host population is roughly equally split between high- and low-yield hosts (% $S_{L}\approx 0.5$, where % $S_{L}=s_{L}(t)/(s_{H}(t)+s_{L}(t))$) and equal to zero when the host population is entirely high- or low-yield hosts. In the case where the high-yield host is slightly more productive from the perspective of the parasite ($R_{eH}>R_{eL}$), $c_{H}=0.1,c_{L}=0,y_{H}=0.1,y_{L}=0.2$, while in the case where the high-yield host is much more productive from the perspective of the parasite ($R_{eH}\gg R_{eL}$), $c_{H}=1,c_{L}=0,y_{H}=0.1,y_{L}=1$. For all cases: $x=0.5,\lambda =50,\delta =0.02,\gamma =0.6,\rho =10^{-2},\epsilon =0.25$.

In line with previous work [18–21], the first model demonstrates that transmission dispersion increases as the difference in host quality increases (figure 1). As expected, transmission dispersion is equal to zero when the host population is composed entirely of low-yield or high-yield hosts. The variance to mean ratio is high when high-yield hosts contribute more infections than low-yield hosts.

### 2.2. A model to study how parasite adaptation impacts transmission dispersion

**Figure 2.**
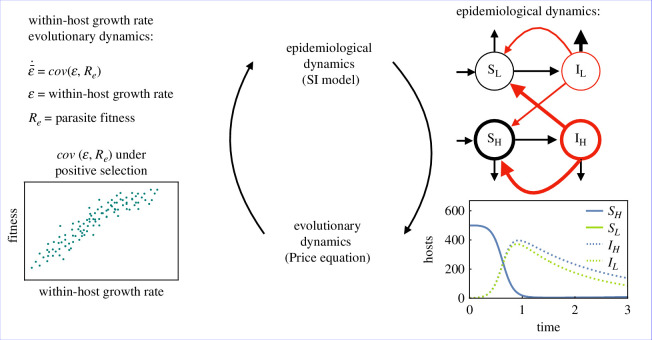
The modelling framework follows the epidemiological dynamics of the host population (using a SI model [22]) and the evolutionary dynamics of the parasite within-host parasite growth rate (using the Price equation [23]). The epidemiological dynamics impact selection on the within-host parasite growth rate. The value of the within-host parasite growth rate impacts how quickly infected hosts transmit the parasite and die from the infection, thus impacting the epidemiological dynamics. The form of the Price equation used here ignores the impact of mutation. The plot in the bottom left shows an example of positive selection on the within-host growth rate as the trait is positively correlated with fitness. The plot in the bottom right shows an example of the epidemiological dynamics for high- and low-yield susceptible and infectious hosts.

In this next section, we outline a model to study how parasite adaptation impacts transmission dispersion over time. To do so, we use a modelling framework that was developed in [12] that follows the epidemiological and evolutionary dynamics of a host–parasite system. In this framework, the epidemiological dynamics are linked to the evolutionary dynamics through the Price equation and thus the impact of parasite adaptation on epidemiological dynamics is explicitly considered and vice versa (figure 2). To get at our question, we use an extension of this original model that considers a heterogeneous host population originally developed to investigate the evolutionary dynamics of parasites adapting to partially vaccinated host populations [24]. In our case, we consider the evolution of a polymorphic parasite population infecting a heterogeneous host population in which the number of infections resulting from each host type can vary due to predetermined biological factors. The trait under selection in this study is the within-host growth rate of the parasite (ϵ).

Following [12], this study uses a form of the Price equation that ignores the impact of mutation to track the mean within-host parasite growth rate (ϵ) in the parasite population in each host type:


(2.7*a*) $$\frac{d{\bar{\epsilon }}_{H}}{dt}=cov(\epsilon ,r_{HH})+\frac{i_{L}}{i_{H}}({\bar{r}}_{LH}({\bar{\epsilon }}_{L}-{\bar{\epsilon }}_{H})+cov(\epsilon ,r_{LH})),$$

(2.7*b*) $$\frac{d{\bar{\epsilon }}_{L}}{dt}=cov(\epsilon ,r_{LL})+\frac{i_{H}}{i_{L}}({\bar{r}}_{HL}({\bar{\epsilon }}_{H}-{\bar{\epsilon }}_{L})+cov(\epsilon ,r_{HL})),$$

where $r_{jk}$ terms specify the *per capita* rate of production of new infections in host type k from host type j (i.e. fitness). The covariance between the within-host growth rate and fitness for each transmission scenario is $cov(\epsilon ,r_{jk})$. The $r_{jk}$ terms are given by


(2.8*a*) $$\begin{array}{lrlr} & & r_{HH}={\bar{\beta }}_{H}s_{H}(t)-(\delta +\gamma +{\bar{\alpha }}_{H}), & \end{array}$$

(2.8*b*) $$\begin{array}{lrlr} & & r_{HL}={\bar{\beta }}_{L}s_{H}(t), & \end{array}$$

(2.8*c*) $$\begin{array}{lrlr} & & r_{LL}={\bar{\beta }}_{L}s_{L}(t)-(\delta +\gamma +{\bar{\alpha }}_{L}), & \end{array}$$

(2.8*d*) $$\begin{array}{lrlr} & & r_{LH}={\bar{\beta }}_{H}s_{L}(t). & \end{array}$$

The first term in equations (2.7*a*) and (2.7*b*) describes the impact of selection on ${\bar{\epsilon }}_{H}$ and ${\bar{\epsilon }}_{L}$ from infections that pass exclusively within one host type (e.g. $i_{H}$ infects $s_{H}$). The second and third terms in equations (2.7*a*) and (2.7*b*) describe the impact of transmission between host types (e.g. $i_{H}$ infects $s_{L}$) and are thus weighted by the relative sizes of the two host type populations. The second term in equations (2.7*a*) and (2.7*b*) expresses the impact transmission between host types has on ${\bar{\epsilon }}_{H}$ and ${\bar{\epsilon }}_{L}$ in the absence of selection. This term will have an impact on trait values when ${\bar{\epsilon }}_{H}$ and ${\bar{\epsilon }}_{L}$ differ. The third term in equations (2.7*a*) and (2.7*b*) accounts for any selection that occurs during the transmission process between host types, e.g. parasites with high ϵ enjoy high transmission and are over-represented among strains that infect $s_{L}$ from $i_{H}$. This term accounts for the fact that transmission between host types can impact ϵ trait values even if ${\bar{\epsilon }}_{H}$ and ${\bar{\epsilon }}_{L}$ are equal. Equations (2.7*a*) and (2.7*b*) can be expanded by assuming $cov(\epsilon ,r_{jk})\approx var_{j}(\epsilon )(dr/d\epsilon )$, which yields


$$\begin{array}{rl} & \frac{d{\bar{\epsilon }}_{H}}{dt}=var_{H}(\epsilon )\left(\frac{d\beta_{H}}{d\epsilon }s_{H}(t)-\frac{d\alpha_{H}}{d\epsilon }\right)+\frac{i_{L}(t)}{i_{H}(t)}\left({\bar{\beta }}_{L}s_{H}(t)(\epsilon_{L}-\epsilon_{H})+var_{L}(\epsilon )\frac{d\beta_{L}}{d\epsilon }s_{H}(t)\right),\\ & \frac{d{\bar{\epsilon }}_{L}}{dt}=var_{L}(\epsilon )\left(\frac{d\beta_{L}}{d\epsilon }s_{L}(t)-\frac{d\alpha_{L}}{d\epsilon }\right)+\frac{i_{H}(t)}{i_{L}(t)}\left({\bar{\beta }}_{H}s_{L}(t)(\epsilon_{H}-\epsilon_{L})+var_{H}(\epsilon )\frac{d\beta_{H}}{d\epsilon }s_{L}(t)\right), \end{array}$$

which, using equation (2.2), expands to


(2.9*a*) $$\frac{d{\bar{\epsilon }}_{H}}{dt}=var_{H}(\epsilon )(\rho x\epsilon_{H}^{x-1}s_{H}(t)-y_{H})+\frac{i_{L}(t)}{i_{H}(t)}({\bar{\beta }}_{L}s_{H}(t)(\epsilon_{L}-\epsilon_{H})+var_{L}(\epsilon )\rho x\epsilon_{L}^{x-1}s_{H}(t)),$$

(2.9*b*) $$\frac{d{\bar{\epsilon }}_{L}}{dt}=var_{L}(\epsilon )(\rho x\epsilon_{L}^{x-1}s_{L}(t)-y_{L})+\frac{i_{H}(t)}{i_{L}(t)}({\bar{\beta }}_{H}s_{L}(t)(\epsilon_{H}-\epsilon_{L})+var_{H}(\epsilon )\rho x\epsilon_{H}^{x-1}s_{L}(t)).$$

## 3. Results

The goal of this study is to determine how parasite adaptation to heterogeneous host populations impacts transmission dispersion. That is, does parasite adaptation skew the contribution that infections from each host type make to parasite fitness (measured as an increase in *vmr*($R_{e}$))? To do so, we use a mathematical modelling framework that follows epidemiological dynamics coupled to the evolutionary dynamics of the parasite trait under study: the within-host growth rate (ϵ). The framework differs from adaptive dynamics in that the epidemiological and evolutionary dynamics proceed simultaneously such that the epidemiological dynamics need not be at an equilibrium for new parasite strains to emerge (i.e. no timescale separation between the epidemiological and evolutionary dynamics). Our approach thus allows us to study how parasite adaptation impacts transmission dispersion before the epidemiological dynamics have settled to an equilibrium. The epidemiological dynamics follow susceptible and infectious host densities (equations (2.1*a*)–(2.1*d*)), which determine parasite reproductive fitness (measured as $R_{e}$, equation (2.4)) and drive the adaptation of the within-host growth rate (equations (2.7*a*) and (2.7*b*)). The epidemiological and evolutionary dynamics are linked such that changes in the within-host growth rate impact the transmission and virulence of infected hosts and thus the epidemiological dynamics. The host population is heterogeneous as there are two distinct host types: (i) a high-yield host that has high transmission and/or low virulence following infection such that parasite reproductive fitness from these hosts is high and (ii) a low-yield host that has low transmission and/or high virulence following infection such that parasite reproductive fitness from these hosts is low. We predict how different host population compositions (e.g. proportion of low-yield hosts, differences in transmission set points) impact parasite evolution, which changes the reproductive fitness of the parasite from both host types and thus the relative contribution both hosts make to parasite fitness, measured as transmission dispersion (equation (2.5)). The model predicts that transmission dispersion is highest when the host population is mostly composed of low-yield hosts and when the difference in quality between the two host types is large.

### 3.1. How transmission dispersion changes over the course of an epidemic

**Figure 3.**
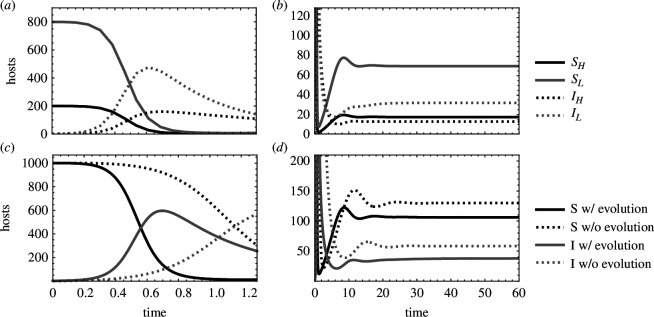
Epidemiological dynamics of a heterogeneous host population early (*a*,*c*) and late (*b*,*d*), with (*a*,*b*) and without evolution (*c*,*d*). (*a*,*b*) The density of susceptible high-yield ($S_{H}$) and low-yield hosts ($S_{L}$) and infectious high-yield ($I_{H}$) and low-yield hosts ($I_{L}$). (*c*,*d*) The total density of susceptible (S) and infectious ($I_{H}$) hosts with and without parasite evolution. In the absence of parasite evolution, the parasite within-host growth rate (ϵ) is set to 0.25 and does not change. Note that high- and low-yield susceptible host densities are identical when they have equal proportions in the host population (p=0.5 in equations (2.1*a*)–(2.1*d*)). $c_{H}=1,c_{L}=0.1,y_{H}=0.1,y_{L}=1,x=0.5,\lambda =50,\delta =0.02,\gamma =0.6,\rho =10^{-2},var_{H}(\epsilon )=var_{L}(\epsilon )=1$.

In order to determine how transmission dispersion changes over time during an epidemic, we first examined how the epidemiological dynamics develop over time. The model shows that susceptible host density is high early in an epidemic and quickly drops as hosts become infected (figure 3*a*). Infected host density peaks relatively early in an epidemic and then drops before eventually rebounding and settling to an endemic equilibrium (figure 3*b*). Infected host density drops as the influx of new infections decreases from the drop in susceptible host availability and as hosts recover and die from the infection. Low-yield infected hosts maintain lower densities than high-yield infected hosts when they experience higher virulence than high-yield hosts. Parasite adaptation changes the dynamics that occur in the absence of evolution by driving an earlier decrease in susceptible host density (figure 3*c*) and maintaining lower equilibrium host densities (figure 3*d*).

**Figure 4.**
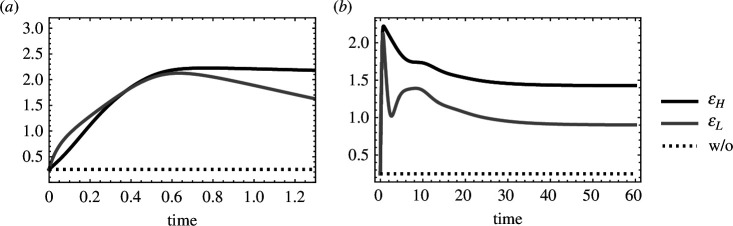
Evolutionary dynamics of the within-host parasite growth rate (ϵ) early (*a*) and late (*b*). The trait value of the within-host parasite growth rate in high-yield ($\epsilon_{H}$) and low-yield hosts ($\epsilon_{L}$). Dotted black line shows the value of the within-host growth rate in the absence of adaptation (ϵ=0.25). $c_{H}=1,c_{L}=0.1,y_{H}=0.1,y_{L}=1,x=0.5,\lambda =50,\delta =0.02,\gamma =0.6,\rho =10^{-2},var_{H}(\epsilon )=var_{L}(\epsilon )=1$.

To study how parasite evolution impacts transmission dispersion throughout an epidemic, we looked at how the trait under selection—the within-host growth rate (ϵ)—changes over time. To understand these dynamics, it is necessary to understand the two selective forces that act on the within-host growth rate: (i) the susceptible host density selects for increased within-host growth rate—with strength proportional to susceptible host density—and (ii) virulence selects for decreased ϵ—with strength proportional to the rate that virulence increases as ϵ increases ($y_{i}$). Early in an epidemic, there is strong selection for high ϵ as susceptible hosts are abundant (figures 3c and 4a). Selection for high ϵ is approximately the same in both host types during this period ($\epsilon_{H}\approx \epsilon_{L}$). As susceptible hosts are depleted, selection for increased ϵ disappears. Low ϵ is then adaptive as negative selection from the cost of virulence outweighs the weak positive selection from the few remaining susceptible hosts (figures 3c and 4a). Selection for higher ϵ resumes as the susceptible host population is replenished and continues until the epidemiological and evolutionary dynamics reach an equilibrium. Note that the extent that $\epsilon_{H}$ and $\epsilon_{L}$ trait values diverge while approaching the evolutionary equilibrium increases as the difference in virulence between hosts increases (this can also be predicted from equations (2.9*a*) and (2.9*b*)).

**Figure 5.**
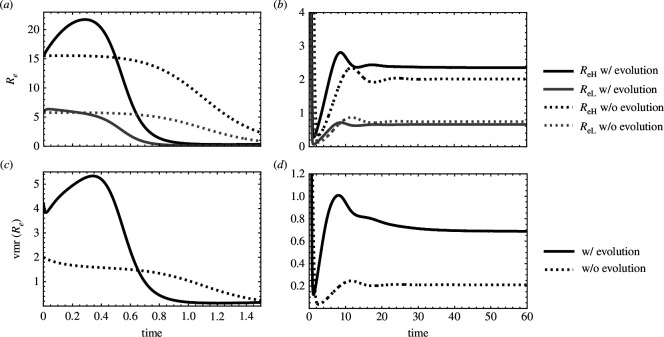
Parasite adaptation drives higher parasite fitness and transmission dispersion. Both parasite fitness and transmission dispersion are highest early in epidemics when susceptible host density is also high. Parasite fitness of high- and low-yield hosts ($R_{eH},R_{eL}$) and transmission dispersion ($vmr(R_{e})$) over time. $c_{H}=1,c_{L}=0.1,y_{H}=0.1,y_{L}=1,x=0.5,\lambda =50,\delta =0.02,\gamma =0.6,\rho =10^{-2},var_{H}(\epsilon )=var_{L}(\epsilon )=1$.

We next studied how the dynamics of parasite fitness ($R_{e}$) and transmission dispersion ($vmr(R_{e})$) are determined by both the epidemiological dynamics and the evolutionary dynamics of the within-host growth rate (figure 5). Parasite fitness in both high- and low-yield hosts is high early in epidemics regardless of parasite adaptation as susceptible host density is high ($R_{eH},R_{eL}$ in figures 3*c* and 5*a*). Parasite adaptation increases the relative fitness from high- and low-yield hosts by driving more new infections from high-yield hosts compared with low-yield hosts (figure 5*a*). Parasite adaptation can thus result in increased transmission dispersion by increasing parasite fitness from high-yield hosts more than low-yield hosts. The early peak in parasite fitness and transmission dispersion is followed by a dip when susceptible hosts are rare (figures 3*c* and 5*a*). Transmission dispersion is also low when hosts are rare as parasite fitness is low for infections of both host types (i.e. $R_{eH}$ and $R_{eL}$ are both low, which drives low $vmr(R_{e})$; figure 5*c*). Parasite fitness and transmission dispersion both rise again as susceptible host abundance increases (figure 5*b*,*d*). At equilibrium, transmission dispersion is lower compared with early in an epidemic as host densities are relatively low (figures 3*d* and 5*a*).

To explore the impact of within-host growth rate variance on transmission dispersion, we conducted additional simulations detailed in appendix A. These simulations reveal how phenotypic variance influences the speed and extent of parasite adaptation during epidemics. Our results indicate that high phenotypic variance in the within-host growth rate significantly accelerates parasite adaptation and increases early transmission dispersion (appendix A, figure 8).

### 3.2. Impact of host composition on transmission dispersion

**Figure 6.**
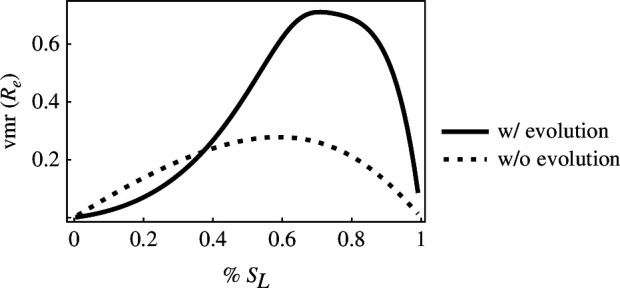
Transmission dispersion is highest when parasites adapt to host populations that are mostly composed of low-yield hosts. The variance-to-mean ratio of $R_{e}(t)$ at the endemic equilibrium as the percentage of susceptible low-yield hosts ($s_{L}$) in the system varies. $c_{H}=1,c_{L}=0.1,y_{H}=0.1,y_{L}=1,x=0.5,\lambda =50,\delta =0.02,\gamma =0.6,\rho =10^{-2},var_{H}(\epsilon )=var_{L}(\epsilon )=1$.

**Figure 7.**
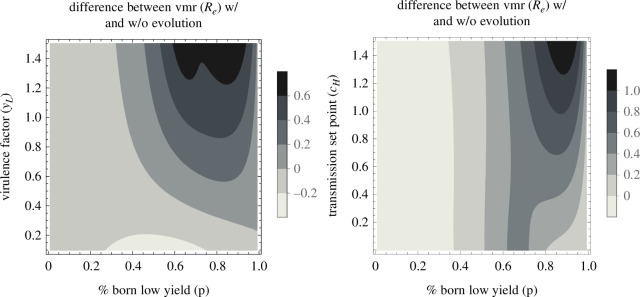
The difference between transmission dispersion ($vmr(R_{e})$) with and without parasite evolution is greatest when most hosts are born low yield (high p) and either high-yield hosts are much higher quality than low-yield hosts from the parasite's perspective ($y_{L}\ll y_{H},c_{H}\gg c_{L}$). $y_{H}=0.1,y_{L}=1,c_{H}=0.5,c_{L}=0$. All other parameters are the same as in figure 3.

To determine how host heterogeneity impacts the extent to which parasite adaptation increases transmission dispersion, we studied how transmission dispersion changes when parasites evolve to different host compositions. The model predicts that the composition of the host population impacts the extent to which parasite adaptation increases transmission dispersion. For example, host populations that are mostly composed of low-yield hosts select for parasites that drive high transmission dispersion (figure 6). Parasites infecting and adapting to host populations with only a few high-yield hosts cause high transmission dispersion as the small proportion of high-yield hosts are responsible for an outsized proportion of new cases. Furthermore, parasite adaptation drives larger increases in transmission dispersion as the difference in quality between high- and low-yield hosts increases (figure 7). Differences in both transmission and virulence can drive increased $vmr(R_{e})$: transmission dispersion is high when low-yield hosts have lower transmission ($c_{H}>c_{L}$) and/or higher virulence ($y_{H}<y_{L}$) compared with high-yield hosts (figure 7). Appendix B contains further plots that show the relationship between host composition and the remaining parameters in the model. These figures demonstrate that parameters that impact host quality have the largest impact on transmission dispersion. Overall, the relationships between transmission dispersion and host composition are robust to shifts in the parameter ranges considered.

## 4. Summary and discussion

This study provides a framework for predicting how parasite adaptation impacts transmission dispersion for emerging and re-emerging infectious diseases. The model predicts that parasite adaptation to heterogeneous host populations can result in increased transmission dispersion. Parasite adaptation drives the greatest increases in transmission dispersion when host populations are composed of high-yield hosts that have higher transmission and lower virulence compared with low-yield hosts. The results predict that parasite adaptation to heterogeneous host populations drives the evolution of high transmission dispersion of parasites early in epidemics. Furthermore, parasite adaptation can maintain increased transmission dispersion at endemic equilibria.

The results of the current study strengthen the idea that differences in host transmission can drive transmission dispersion but also predict that differences in virulence can increase transmission dispersion by impacting parasite adaptation. That is, epidemiological studies have previously shown that differences in transmission potential among hosts (often measured as parasite load) are a source of heterogeneity that is associated with increased transmission dispersion [25]. This study suggests that heterogeneity in host transmission potential can also indirectly increase transmission dispersion by selecting for parasites that drive more infections from higher yield hosts than lower yield hosts. Thus, given that these results suggest the possibility that differences in (host) virulence can select for parasites that enhance transmission dispersion, more effort should be made to experimentally disentangle the relationship between virulence and transmission dispersions.

The variation of virulence across host types is predicted to determine whether parasite adaptation drives increased transmission dispersion; however, virulence manifests in many different ways in nature. An obvious question is thus how different virulence modes could impact the predictions made here. Virulence in this study is modelled as an increase in host mortality following infection that increases with the parasite within-host growth rate. Parasites can enjoy a high growth rate on high-yield hosts that are more tolerant to infection (i.e. high-yield hosts that do not experience high mortality despite being infected by parasites with high growth rates). Transmission dispersion can result from some hosts dying more quickly than others. Thus, alternative forms of virulence that also vary between hosts and shorten the duration of the infection could lead to similar phenomena as predicted here. For example, hosts could vary in the severity of the symptoms they experience post-infection (e.g. lethargy). Hosts experiencing severe symptoms may then decrease contact with other hosts and thus decrease the likelihood that the infection is spread. Similarly, hosts could vary in how quickly they experience symptoms after becoming infectious. Hosts that quickly experience symptoms may also be less likely to spread the infection through decreased contact with other hosts. Finally, the results of the current study could apply to the heterogeneous distribution of treatment against infection that decreases virulence such that increased transmission dispersion could be adaptive if transmission is not impacted.

**Figure 8.**
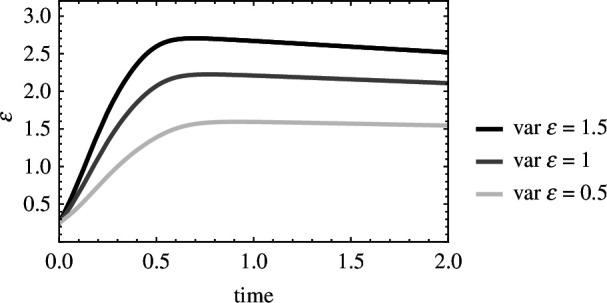
Phenotypic variance in the population of the within-host growth rate (var ϵ) controls how quickly adaptation occurs. Plots show the dynamics of within-host growth rate (ϵ) adaptation for different values of var ϵ and demonstrate that ϵ adapts more quickly and reaches higher values when var ϵ is high. All parameters are the same as in figure 3.

The phenotypic variance in the within-host parasite growth rate impacts transmission dispersion by determining selection strength (figure 8). Low variance in the within-host growth rate constrains parasite adaptation by limiting the range of trait values that natural selection can act upon, which keeps the within-host growth rate and transmission dispersion low. Conversely, high variance in the within-host growth rate selects for high within-host growth rates in high-yield hosts but low within-host growth rates in low-yield hosts and thus slightly lower transmission dispersion compared to intermediate variance. The current study assumes that the variance in the within-host growth rate is the same in both host types; however, in nature, high-yield hosts would likely support higher variance as they are infected for longer periods of time and maintain higher parasite loads. Relaxing this assumption and assuming that selection on the within-host growth rate in low-yield hosts is subject to low variance would likely drive higher transmission dispersion as selection for low within-host growth rates in low-yield hosts would be weaker than selection for high within-host growth rates in high-yield hosts.

Often highly infectious hosts experience high virulence due to the burden of carrying large parasite loads [26,27]. However, observations of hosts that tolerate high parasite loads while experiencing little or no increase in virulence are also common [5,28]. This study combines some of both ideas by assuming that virulence increases as the within-host parasite growth rate increases but also assumes that a subset of hosts tolerates those increases in growth rate well. Further, this study assumes that the hosts that experience low virulence also transmit at a higher rate. In this way, we have focused on the two extremes in the disease ecology literature: hosts that transmit a lot because of the combination of low virulence and high transmission, and hosts that only transmit a little because they simultaneously have high virulence and low transmission. In reality, host populations will also be composed of intermediate host types, e.g. hosts that have high virulence and high transmission, hosts that are resistant to infection naturally or through treatments that result in low virulence and low transmission. We focused on the two extreme host cases because it was most likely to enhance the impact of heterogeneity on the evolution of increased parasite transmission dispersion. Less extreme forms of host heterogeneity have been considered in other studies. For example, Gog *et al.* [29] assumed that a proportion of the host population is more vulnerable to the infection but mixes less with other hosts while all other hosts are less vulnerable to the infection but mix more readily. Similarly, [30] studied how high and low contact rates as well as high and low vulnerability to infection following vaccination impacted the speed that parasites evolve. Parasites that adapt to these more intermediate host types will likely drive more modest increases in transmission dispersion compared to the host populations studied here. Nevertheless, future work should study the effect of parasite adaptation to intermediate host types on transmission dispersion.

A key question is how common the host compositions studied here are in nature. Empirical studies on host heterogeneity tend to focus on one trait: either transmission (often measured as parasite load or contact rate) or virulence (this is measured in many ways, e.g. symptoms, fertility, death rate). However, one study that is relevant to the assumptions of this model showed that the distribution of SARS-CoV-2 viral loads among symptomatic/pre-symptomatic and asymptomatic cases were similar [25]. In other words, viral load distributions are not clearly associated with host virulence and thus a subset of asymptomatic hosts could have very high viral loads. Thus, while there is some evidence that high- and low-yield hosts similar to those modelled in this study exist, the exact compositions of host populations are not well documented. That is, the percentages of hosts that are high yield and low yield, as well as the distributions of intermediate traits, such as transmission found in host populations, are often not known. Thus, more empirical research is necessary to determine how the compositions of host traits relevant to parasite spread vary in host populations.

The predictions made in this study should be tested experimentally. Successfully validating theory requires controlled experiments where the factor of interest can be manipulated to compare empirical results to theoretical predictions. Experiments to test whether parasite adaptation to heterogeneous host populations can drive increased transmission dispersion will require a disease system with two host types that differ in transmission and/or virulence. Many disease systems fit one of these criteria in that host types have been identified that are capable of causing a disproportionate number of new infections either through increased shedding of the parasite [31–35] or through decreased virulence (e.g. long infectious periods) [36,37]. One disease system that could be well suited to test the predictions made in this study is *Daphnia magna* and its bacterial parasite, *Pasteuria ramosa*. The *Daphnia*–*Pasteuria* system is ideal in that both spore load (i.e. transmission potential) and virulence (measured as reductions in post-infection lifespan or fecundity) can vary between males and females [38] and across age classes [39]. Thus it may be possible to use existing *Daphnia* lines that meet the high- and low-yield host classifications used in this study and test the predictions made here.

Most new infections are transmitted from relatively few infected individuals. This increased transmission dispersion is largely attributed to differences among hosts; thus, most research to date has focused on the importance of variability in host populations. The current study presents an additional evolutionary mechanism that could enhance this phenomenon whereby parasite specialization on highly infectious hosts comes at the cost of transmitting less effectively from less-infectious hosts. The model predicts that, in this way, parasite adaptation can further skew transmission events so that most new infections are transmitted from a minority of infectious hosts. Further, this study presents a framework for predicting how parasite adaptation determines transmission dispersion for emerging and re-emerging infectious diseases.

## Figures and Tables

**Figure 9.**
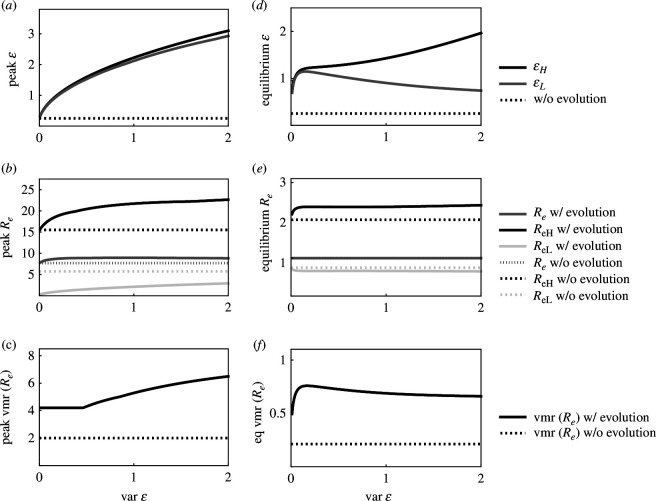
Variance in the within-host growth rate (var ϵ) impacts parasite adaptation and transmission dispersion. (*a*–*c*) Plots show how var ϵ impacts peak within-host growth rates (ϵ), $R_{e}$ and transmission dispersion (vmr$\left(R_{e}\right)$) that occur early in the epidemic (see figure 5). (*d*–*f*) Plots show how var ϵ impacts the within-host growth rate (ϵ), $R_{e}$ and transmission dispersion (vmr$\left(R_{e}\right)$) at the endemic equilibrium (see figure 5). All parameters are the same as in figure 3.

**Figure 10.**
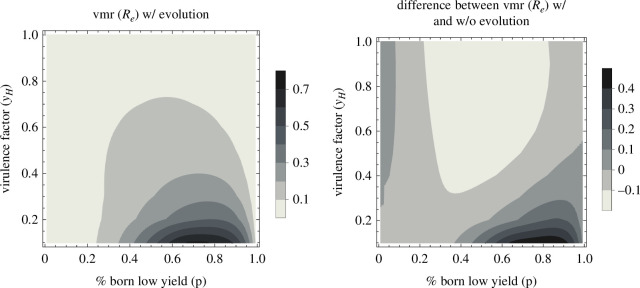
Transmission dispersion (vmr$\left(R_{e}\right)$) is greatest when most hosts are born low yield and when virulence is low in high-yield hosts (small $y_{H}$). Low virulence in high-yield hosts results in a large difference in transmission potential between the high- and low-yield hosts. The difference in vmr$\left(R_{e}\right)$ with and without parasite evolution is also the greatest when most hosts are born low yield (high p) and virulence is low in high-yield hosts (small $y_{H}$). Note that the baseline value for $y_{H}$ in the main text is 0.1. All other parameters are the same as in figure 3.

**Figure 11.**
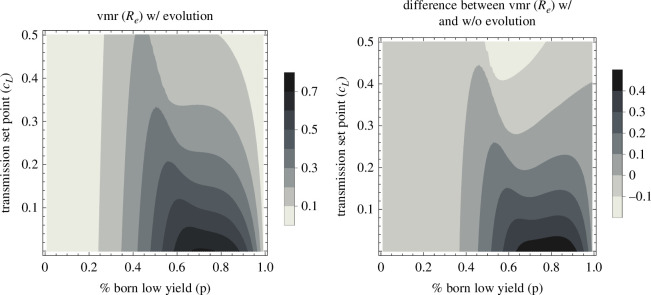
Transmission dispersion (vmr$\left(R_{e}\right)$) is greatest when most hosts are born low yield and when the transmission set point is low in low-yield hosts (small $c_{L}$). A low transmission set point in low-yield hosts results in a large difference in transmission potential between the high- and low-yield hosts. The difference in vmr$\left(R_{e}\right)$ with and without parasite evolution is also the greatest when most hosts are born low yield (high p) and the transmission set point is low in low-yield hosts (small $c_{L}$). Note that the baseline value for $c_{L}$ in the main text is 0.1. All other parameters are the same as in figure 3.

**Figure 12.**
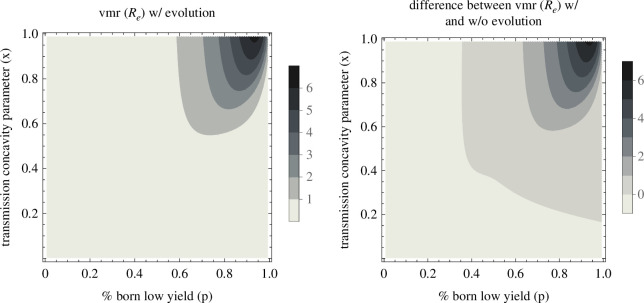
Transmission dispersion (vmr$\left(R_{e}\right)$) is greatest when most hosts are born low yield and when the transmission concavity parameter (x) is large. Transmission increases from increased within-host growth rates (ϵ) approach a linear increase as the concavity parameter in the transmission function approaches 1. Thus, large x results in a large difference in transmission potential between the high- and low-yield hosts. The same holds true for the difference in vmr$\left(R_{e}\right)$ with and without parasite evolution. Note that the baseline value for x in the main text is 0.5. All other parameters are the same as in figure 3.

**Figure 13.**
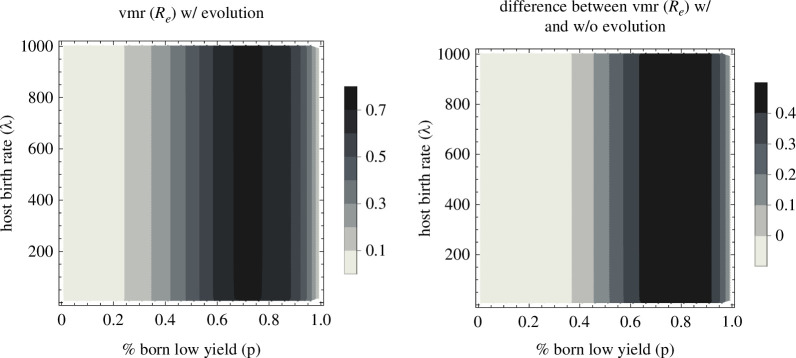
Transmission dispersion (vmr$\left(R_{e}\right)$) is greatest when most hosts are born low yield. The rate that new hosts are born (λ) has no impact on transmission dispersion in this parameter range. The same holds true for the difference in vmr$\left(R_{e}\right)$ with and without parasite evolution. We also investigated how changes in λ impact the epidemiological dynamics but do not include these results in the text because they were negligible. Note that the baseline value for λ in the main text is 50. All other parameters are the same as in figure 3.

**Figure 14.**
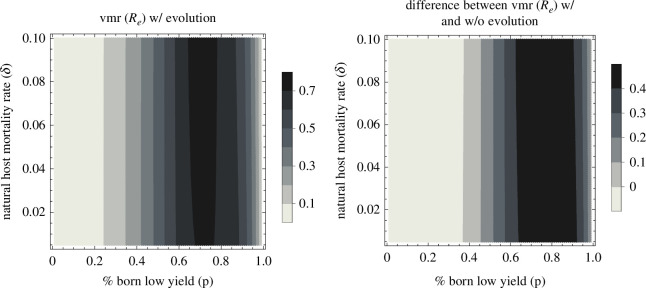
Transmission dispersion ($vmr(R_{e})$) is greatest when most hosts are born low yield. The natural host mortality rate (δ) has no impact on transmission dispersion in this parameter range. The same holds true for the difference in vmr$\left(R_{e}\right)$ with and without parasite evolution. Note that the baseline value for δ in the main text is 0.02. All other parameters are the same as in figure 3.

**Figure 15.**
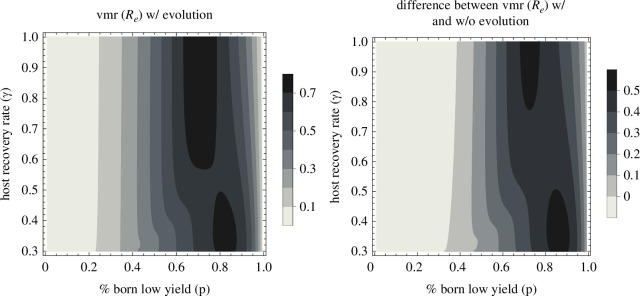
Transmission dispersion (vmr$\left(R_{e}\right)$) is greatest when most hosts are born low yield. The host recovery rate (γ) has a small impact on transmission dispersion such that intermediate values for high p decrease transmission dispersion slightly. The same holds true for the difference in vmr$\left(R_{e}\right)$ with and without parasite evolution. Note that the baseline value for γ in the main text is 0.6. All other parameters are the same as in figure 3.

**Figure 16.**
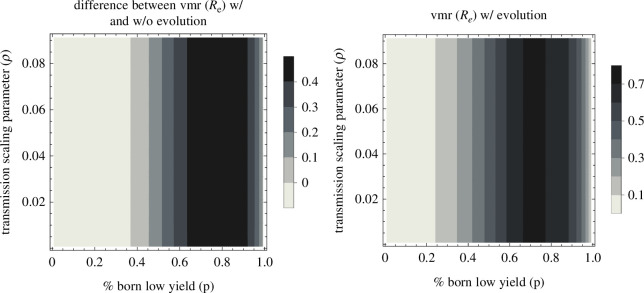
Transmission dispersion (vmr$\left(R_{e}\right)$) is greatest when most hosts are born low yield. The transmission scaling parameter (ρ) has no impact on transmission dispersion in this parameter range. The same holds true for the difference in vmr$\left(R_{e}\right)$ with and without parasite evolution. Note that the baseline value for ρ in the main text is $10^{-2}$. All other parameters are the same as in figure 3.

## Data Availability

Code is available on Zenodo [40].

## Appendix A. Parasite within-host growth rate variance impacts transmission dispersion

This appendix investigates the impact of variance in the within-host growth rate on transmission dispersion. The phenotypic variance of the within-host growth rate in the parasite population (var ϵ) controls how quickly parasite adaptation occurs (figure 8). Early in an epidemic, high phenotypic variance drives increased peak within-host growth rates as parasites can quickly adapt to exploit high host densities (figure 8). When phenotypic variance is low, parasite adaptation proceeds more slowly such that host densities drop, reducing selection pressure for high within-host growth rates before high trait values evolve. High phenotypic variance also drives high transmission dispersion (vmr($R_{e}$)) early in the epidemic through the high values of the within-host growth that result in larger increases in parasite fitness from high-yield hosts ($R_{eH}$) relative to fitness from low-yield hosts ($R_{eL}$) (figure 9*a–c*). Conversely, low phenotypic variance prevents high within-host growth rates from evolving and thus maintains low transmission dispersion by keeping $R_{eH}$ and $R_{eL}$ relatively close to one another.

The impact of phenotypic variance on parasite adaptation differs at the endemic equilibrium compared to early in an epidemic: intermediate phenotypic variance maximizes equilibrium transmission dispersion (figure 9*f*). Similar to early in an epidemic, increasing phenotypic variance increases the within-host growth rate and transmission dispersion for small values of phenotypic variance (figure 9*d,f*). However, further increases in phenotypic variance decrease the within-host growth rate in low-yield hosts ($\epsilon_{L}$) while maintaining high within-host growth rates in high-yield hosts ($\epsilon_{H}$) (figure 9*d*). When phenotypic variance is low, selection dominates to increase $\epsilon_{L}$ and $\epsilon_{H}$ to prioritize increasing fitness on high-yield hosts at the cost of decreased fitness on low-yield hosts (figure 9*d*). Conversely, high phenotypic variance results in within-host growth rate specialization in the two host types, which maintains high reproductive fitness in both hosts and simultaneously decreases transmission dispersion (figure 9*d,f*).

## Appendix B

This appendix contains plots that show how the impact of parasite adaptation on transmission dispersion ($vmr(R_{e})$) changes as the per cent of susceptible low-yield hosts that are born into the host population (p) changes along with the other parameters in the evolutionary model (figures 10–16). For each parameter set, we show these results with contour plots of the transmission dispersion with parasite evolution and the difference in transmission dispersion with and without parasite evolution for comparison.

We selected parameter values based on ranges that seemed biologically plausible and led to meaningful dynamics in our simulations. Our goal was to explore the qualitative behaviors and dependencies within this parameter space, rather than to produce exact predictions. Moreover, we found that the core dynamics of the model remained consistent across a range of these values, suggesting the robustness of our findings to parameter variability. Future work could certainly benefit from more precise parameter estimation as relevant data become available.
